# A novel recessive mutation affecting DNAJB6a causes myofibrillar myopathy

**DOI:** 10.1186/s40478-020-01046-w

**Published:** 2021-02-08

**Authors:** Fang-Yuan Qian, Yu-Dong Guo, Juan Zu, Jin-Hua Zhang, Yi-Ming Zheng, Idriss Ali Abdoulaye, Zhao-Hui Pan, Chun-Ming Xie, Han-Chao Gao, Zhi-Jun Zhang

**Affiliations:** 1 Department of Neurology, Affiliated ZhongDa Hospital, School of Medicine, Research Institution of Neuropsychiatry, Southeast University, Nanjing, Jiangsu China; 2 Department of Orthopedic, Affiliated ZhongDa Hospital, School of Medicine, Southeast University, Nanjing, Jiangsu China; 3 Department of Nephrology, Shenzhen Longhua District Central Hospital, Guangdong Medical University, Shenzhen, China; 4 Department of Neurology, Peking University First Hospital, Beijing, China; 5 Key Laboratory of Developmental Genes and Human Disease, Southeast University, Nanjing, Jiangsu China

**Keywords:** DNAJB6a, Myofibrillar myopathy, Novel, Homozygous mutation, Human, Mice

## Abstract

Mutations in the *DNAJB6* gene have been identified as rare causes of myofibrillar myopathies. However, the underlying pathophysiologica mechanisms remain elusive. DNAJB6 has two known isoforms, including the nuclear isoform DNAJB6a and the cytoplasmic isoform DNAJB6b, which was thought to be the pathogenic isoform. Here, we report a novel recessive mutation c.695_699del (p. Val 232 Gly fs*7) in the *DNAJB6* gene, associated with an apparently recessively inherited late onset distal myofibrillar myopathy in a Chinese family. Notably, the novel mutation localizes to exon 9 and uniquely encodes DNAJB6a. We further identified that this mutation decreases the mRNA and protein levels of DNAJB6a and results in an age-dependent recessive toxic effect on skeletal muscle in knock-in mice. Moreover, the mutant DNAJB6a showed a dose-dependent anti-aggregation effect on polyglutamine-containing proteins in vitro. Taking together, these findings reveal the pathogenic role of DNAJB6a insufficiency in myofibrillar myopathies and expand upon the molecular spectrum of *DNAJB6* mutations.

**Electronic supplementary material:**

The online version of this article (10.1186/s40478-020-01046-w) contains supplementary material, which is available to authorized users.

## Introduction

Myofibrillar myopathies (MFMs) are a subset of progressive and progressive neuromuscular diseases, which also affect the cardiac and/or respiratory muscles [24, 32]. Although the subtypes of MFMs shown the variations in symptoms and onset age, they all share similar histopathological features, including Z-disk streaming and disruption, aggregation of Z-disk proteins, and mitochondrial abnormalities [41, 48]. For example, mutations in many genes can cause MFMs, including *DES*, *FLNC*, *MYOT*, *CRYAB*, *ZASP*, *BAG3*, *FHL1*, *TTN*, *PLEC*, *ACTA1*, and *HSPB8*. Previous studies have observed the localization of their corresponding proteins within the Z-disk [23]. Notably, it has recently been confirmed that the involvement of DNAJB6 results in the initiation of MFMs [17].

**Fig. 1:**
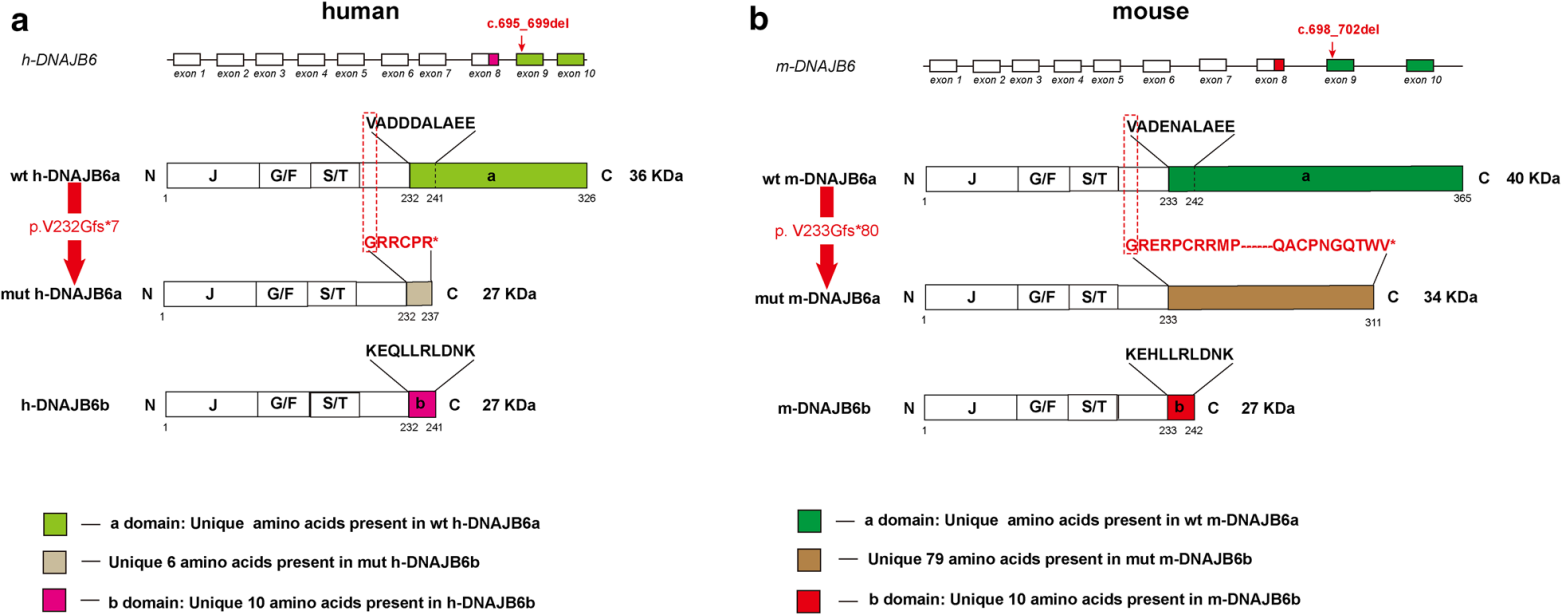
Schematic representation of the *DNAJB6* gene exons, DNAJB6 protein isoforms and the mutation. **a** Sequence comparison of the wildtype DNAJB6a, DNAJB6b and mutant DNAJB6a in human. **b** Sequence comparison of the wildtype DNAJB6a, DNAJB6b and mutant DNAJB6a in mouse. Note that the alternatively spliced C-terminal part of the “a” or “b” region is identified as the marker of the DNAJB6 isoform

DNAJB6, a member of the HSP40 family of co-chaperones, is widely expressed in human and murine tissues with variable levels [6, 40]. It consists of three conserved domains as follows: (i) the N-terminal domain is α-helical and also known as the J-domain; (ii) the G/F domain, rich in glycine/phenylalanine residues, encompasses the majority of disease mutations; (iii) the C-terminal domain contains a serine/threonine (S/T)-rich region [16]. DNAJB6 is expressed as two isoforms, DNAJB6a and DNAJB6b, which differ in the length of their C-terminal region and their cellular localization (Fig. 1) [7]. DNAJB6a is longer and predominantly localizes to the nucleus, whereas DNAJB6b misses the “a” region and exhibits both cytosolic and nuclear localization in tissue culture cells [7]. Both the isoforms have demonstrated a protective mechanism that plays a crucial role in protein folding disorders, such as Huntington’s, Alzheimer’s, and Parkinson’s diseases, through the inhibition of polyglutamine (polyQ)-containing proteins, amyloid-β42 (Aβ42), and alpha-synuclein aggregation [10, 15, 16, 26]. In 2012, a mutation in DNAJB6 was firstly found to cause MFMs [17]. DNAJB6-related MFM was originally described as late-onset, slowly progressive limb-girdle muscular dystrophy D1 (LGMD D1). However, several mutations can also cause an earlier, more severe, or distal-onset myopathy [14, 17, 28, 30, 35, 45]. Until recently, 18 pathogenic heterozygous mutations have been reported, and all altered amino acids were determined to be localized to the G/F and J domains [31, 38].

To date, the pathological mechanisms of DNAJB6-related MFM are not fully clarified yet. The phenotype of protein accumulations presenting in DNAJB6-mutated muscles indicates the impaired ability to degrade the misfolded proteins. Indeed, studies have shown that all reported disease-causing *DNAJB6* mutations are linked to a reduction in anti-aggregation capacity in vitro [18, 30, 37, 42]. Intriguingly, the cytoplasmic isoform DNAJB6b, was thought to be the principal mediator of MFM pathogenesis [4], whereas the role of DNAJB6a in skeletal muscle remains unclear.

The present study reports a novel homozygous mutation, c.695_699del (p. Val 232 Gly fs*7) in DNAJB6, which is associated with an apparently recessively inherited late onset distal myofibrillar myopathy in a Chinese family. Interestingly, the mutation was found to be located to exon 9, which exclusively causes loss of the “a” region in DNAJB6a (Fig. 1). Nevertheless, this mutation has not been previously reported as the cause of a DNAJB6-related disorders and the function of the “a” region was unknown. Therefore, we utilized cellular and animal models to further investigate the functional consequences of this novel mutation.

## Materials and methods

### Patient selection and evaluation

A Chinese family, which presented with a genetically recessive MFM in combination with dyspnea was studied. The clinical records and pathology were reviewed and those who were available were reevaluated for muscle strength, serum creatine kinase (CK) levels, cardiac examination (including electrocardiogram and echocardiogram), pulmonary function, and nerve conduction and electromyography studies.

### Targeted exome sequencing and bioinformatics analysis

Genomic DNA from peripheral blood leukocytes, derived from the proband (II-5) and his available family members, was extracted using a QIAamp DNA blood midi kit (Qiagen, Hilden, Germany). Targeted sequencing was performed on the proband using a custom panel of 133 genes known or predicted to cause muscular dystrophy or myopathy. The exon regions were specifically enriched using a biotinylated capture probe (MyGenostics, China). The enrichment libraries were sequenced on an Illumina HiSeq 2000 sequencer (Illumina, San Diego, California) using 100-bp paired-end reads.

After sequencing, the raw reads from each sample were sorted by index sequences. The adapter sequences were trimmed using the Cutadapt application (http://code.google.com/p/cutadapt/). SolexaQA software was used to remove low-quality bases (< Q20). Clean reads were aligned to the human reference genome (hg19) using the Burrows-Wheeler Aligner (BWA; ver. 0.7.11) software package [9, 44]. Following alignment, PCR duplicates were removed using the Picard tools MarkDuplicates package (ver. 1.109). Realignment around known indel sites and base quality score recalibration (BQSR) were performed using GATK software (ver. 3.3) [25]. GATK Haplotype Caller software was used to call raw variants. The indels and SNPs were annotated using ANNOVAR [27]. Public versions of the databases dbSNP138, 1000 Genome Project, Exome Sequencing Project, ClinVar, and HGMD [49] were used to screen the variants. Prediction of functional effects was evaluated using PolyPhen-2 and SIFT scores [2, 43]. The 300 in-house Asia database, generated using next-generation sequencing data of DNA from 300 normal Chinese individuals provided by MyGenostics was also used to filter variants. Variants were correlated with patient phenotypes and the results of clinical investigations. All variants were classified in the American College of Medical Genetics and Genomics standards and guidelines [34].

### Confirmation of variants using Sanger sequencing

Sanger sequencing was used to confirm that all variants identified by targeted exome sequencing were present and to confirm familial segregation. Primers were designed using Primer3 software [46] and PCR products were sequenced on an ABI 3730xl DNA Analyzer (Applied Biosystems). The genotype–phenotype co-segregation analysis was performed for all members of the family from those who provided samples.

### Muscle magnetic resonance imaging (MRI)

Lower limb muscle MRI was performed on the proband (II-5) and his available family members using a 3.0T MR scanner (Signa Excite, Siemens, Germany). T2-weighted (TR: 4000–6800 ms; TE: 76–89 ms; slice thickness: 5 mm; interslice gap: 4–6 mm) and T1-weighted imaging (TR: 500–689 ms; TE: 9–20 ms; slice thickness: 4–5 mm; interslice gap: 4–5 mm) was used to identify affected muscles, fatty degenerative changes, and muscle edema.

### Generation of *Dnajb6* c.698_702del knock-in mouse models

The coding sequence (CDS) of human *DNAJB6* (NM_058246) displayed a 90% homology identity with the mouse sequence (NM_001037940). The homologous sequences of *DNAJB6* c.695_699 of the human gene corresponded to *DNAJB6* c.698_702 in the mouse.

*DNAJB6* c.698_702del knock-in (KI) mice were generated using a CRISPR/Cas9 based approach. Briefly, two sgRNAs were designed using a CRISPR design tool (http://www.sanger.ac.uk/) to target either a region upstream or downstream of exon 9 and then screened for on-target activity using a universal CRISPR activity assay (UCA™, Biocytogen Inc, China). A T7 promoter sequence was added to the Cas9 or sgRNA template in vitro using PCR amplification. The gene-targeting vector containing *DNAJB6* (c.698_702del) and two homology arms at the left (875 bp) and right (1527 bp) of each was used as a template to repair the double-strand breaks generated by Cas9/sgRNA. The Cas9 mRNA, sgRNAs, and targeting vector were co-injected into the cytoplasm of one–cell stage fertilized C57BL/6 N eggs. The injected zygotes were transferred into oviducts of Kunming pseudopregnant females to generate F0 mice. F0 mice with the expected genotype confirmed by tail genomic DNA PCR and sequencing were mated with C57BL/6 N mice to establish germline-transmitted F1 heterozygous mice. Homozygote (HOM), heterozygote (HET), and wild type (WT) littermates were obtained by crossing HET lines.

### Mouse behavioral phenotypes

All behavioral experiments were conducted following the recommendation of the Ethical Committee for Laboratory Animals (Southeast University). Motor functions were assessed in the adult female mice at 3, 6, 9, 12 and 18 months of age. The behavioral assessment was performed by an experimenter blind to the mouse genotype. Experiments were performed at the same time and day of each weekday in the same room to control for odors and noises.

#### Hanging test

The maximum hanging duration was tested for each mouse according to the protocols described previously [1]. A metal clothes hanger was constructed with 2 mm diameter wire. The mouse was placed close to the equipment and was asked to grasp the wire using its fore-paws only. The timer was started when the mouse was suspended. The test session ended if the mouse was able to hang for 600 s. Inversely, the test was permitted once more when mice released grip of the wire earlier than 600 s, and a maximum hanging duration was recorded and used for the further analysis.

#### Grip strength test

Four-limb grip strength was measured using a grip strength tester (BIO-G3S, Bioseb, France), in accordance with a previous report [1]. Mice were placed on a grid accessory and pulled firmly backward by the tail to provoke a grip response. The maximum force exerted was recorded when the mice released their grip of the grid. The grid grip test was conducted three times in a row after which the mouse was returned to the cage to rest for at least one minute. The mouse performed five series of pulls, each followed by a short rest, for a total of 15 tests. The final score was determined by calculating the mean of the three highest values of the 15 recorded.

#### Rotarod test

Mice were trained every other day for two weeks to adapt to a rotarod apparatus (LE 8205, Panlab, Spain). After training, motor activity was measured using the apparatus. Briefly, the mice were placed in separated sections on an accelerating rotating rod (4–40 rpm over a 300-s period) and the delay in falling was recorded, as described previously [5]. The test was performed three times for each animal, and the mean duration determined for each group.

#### Treadmill experiments

The maximum duration of running was measured using a treadmill (YLS-12L, Tai Meng Inc., China), as described previously [12]. Three days before the experiment, the mice were acclimated to a motor-driven treadmill operating at 14 m/min at a 0% incline. Mice were forced to run until exhausted (remaining on the electrical shocker plate for five seconds). Electrical stimulation was set at the recommended intensity, as described in the instructions.

### Plasmid constructs

A fragment containing the full-length coding region of human *DNAJB6a* (NM_005494.2) or mutation c.695_699del (p.V232Gfs*7) was cloned into a pcDNA3.1 vector to express the Flag (M2 or GFP)-tagged DNAJB6 wild-type or mutant protein. A huntingtin construct, *pcDNA3.1*-*HttEx1*-*(Q)74*-*hrGFP*, encoding an N-terminal fragment of the huntingtin protein (Htt) with 74 polyglutamine (polyQ) residues fused to hrGFP (HttEx1-(Q)74-hrGFP) (a gift from Dr. Hasholt, Section of Neurogenetics, Department of Cellular and Molecular Medicine, University of Copenhagen, Denmark) was constructed.

### Histochemistry, immunohistochemistry, and immunofluorescence

Frozen muscle sections were processed using routine histochemical staining, including hematoxylin & eosin (H&E), modified Gomori trichrome (MGT), succinate dehydrogenase (SDH), and cytochrome oxidase (COX).

Following fixation of the sections in 4% paraformaldehyde, further immunohistochemical and immunofluorescent analysis was performed on the proband (II-5) using the primary antibodies DNAJB6 (ab96539, Abcam or 2C11-C1, Novus Biologicals), desmin (ab49811, Abcam), p62 (ab109012, Abcam), TDP-43 (10782-2-AP, Proteintech), LC3B (#3868, Cell Signaling Technology) and Dysferlin (ab124684, Abcam).

For immunofluorescence of mouse muscle samples, antibodies against the following proteins were used: DNAJB6 (ab96539, Abcam), desmin (ab49811, Abcam), anti-sarcoglycan (ab49811, Abcam), anti-myosin heavy chain (MyHC) isoforms types I, IIA and IIB (BA-D5, SC-71, and BF-F3 from Developmental Studies Hybridoma Bank). LC3B (#3868, Cell Signaling Technology), TDP-43 (10782-2-AP, Proteintech), p62 (ab109012, Abcam). Immunofluorescence analysis was performed as described previously [30]. Images were recorded using an Olympus FV-1000 confocal microscope (Olympus, Japan). Non-fluorescent images were acquired using an Olympus BX51 microscope (Olympus, USA).

HEK293 cells were cultured on coverslips and the following plasmids transfected into the cells using Lipofectamine 2000 (Invitrogen, Waltham, MA): empty plasmid, wild-type *DNAJB6a* plasmid (WT), varying doses of the mutant *DNAJB6a* and *pcDNA3.1*-*HttEx1*-*(Q)74*-*hrGFP* plasmids. After 48 h, the transfected cells were washed with phosphate-buffered saline (PBS) and fixed in 3.7% paraformaldehyde for 10 min, washed in PTB (1 × PBS, 0.1% triton X-100, 0.1% bovine serum albumin). Images were obtained using a Leica DMIL fluorescent inverted microscope (Leica Microsystems).

### Electron microscopy (EM)

Muscle specimens were fixed in 2.5% glutaraldehyde in 0.1 M cacodylate buffer (pH 7.4, 4 °C, 48 h) and then processed as described previously [30]. Images were acquired using an H-7650 electron microscope (Hitachi - Science & Technology, Berkshire, UK).

### Real-time quantitative polymerase chain reaction (RT-qPCR)

Frozen tissues or HEK293 cell lysates were homogenized in Trizol, and total RNA was precipitated in isopropanol. RNA quality and quantity were verified using a Nanodrop 2000 bioanalyzer (Thermo Scientific). Approximately 4 μg RNA was reverse transcribed to cDNA using a PrimeScript RT kit (Takara). Reactions were performed for 15 min at 37 °C, and then 5 s at 85 °C.

Real-time quantitative polymerase chain reaction (RT-qPCR) was conducted using 10 ng cDNA diluted in the SYBR Green mix (Takara) and then tested using an ABI PRISM 7300 real-time PCR System (Applied Biosystems) with gene-specific primers (details of sequences provided in Additional file 1). Relative gene expression was calculated using the 2 ^−ΔΔCt^ method after normalization to *GAPDH* endogenous gene expression. All quantitative RT-PCR analyses were performed in biological triplicate.

### Western blot (WB) analysis

Muscle samples or HEK293 cell lysates were homogenized in RIPA buffer (50 mM tris, 150 mM NaCl, 1% NP40, 0.5% sodium deoxycholate, and 0.1% sodium dodecyl sulfate) supplemented with protease and phosphatase inhibitors (Roche, Indianapolis, IN, USA). Equal protein concentrations were separated on 12% SDS-PAGE gels and then transferred to nitrocellulose membranes. The membranes were blocked in 5% milk TBST (20 mM tris–HCl, 150 mM NaCl, pH 7.6) containing 0.1% (v/v) tween 20 at room temperature for 1 h. Membranes were probed with primary antibodies in blocking solution overnight at 4 °C. Membranes were labeled with the appropriate HRP-conjugated secondary antibodies and then visualized using enhanced chemiluminescence detection reagents (Millipore, Billerica, MA, USA). The following primary antibodies were used: DNAJB6 (ab198995, Abcam), desmin (ab32362, Abcam), TDP-43 (10782-2-AP, Proteintech), LC3B (#3868, Cell Signaling Technology), p62 (ab109012, Abcam), M2 (#14793, Cell Signaling Technology), GFP (#2555, Cell Signaling Technology), β-actin (#3700, Cell Signaling Technology), and GAPDH (#5174, Cell Signaling Technology). Western blots were quantified using ImageJ software, as described previously [33]. All Western blot analyses were analyzed using biological triplicates (n = 3).

### Filter-trap assay (FTA)

The filter-trap assay was performed as described previously [37]. Briefly, HEK293 cells were transfected with an empty plasmid, wide-type DNAJB6a plasmid, different doses of the mutant DNAJB6a plasmid, and 74Q-HTT. After 48 h, the cells were lysed using 750 μl FTA buffer (10 mM tris–HCl, pH 8.0, 150 mM NaCl, 50 mM dithiothreitol) containing 2% SDS and 1 × Complete protease inhibitor (Roche) through a 27G needle and then sonicated at room temp for 1 min. The cell lysates were heated for 3 min at 98 °C and then 100 μl was filtered with light suction through a 0.2 μm cellulose acetate membrane filter (Whatman GmbH). The filter was washed three times with 300 μl FTA buffer containing 0.1% SDS. The quantity of aggregated huntingtin in the cells was measured using anti-GFP and analyzed as per WB analysis.

## Statistical analysis

Values are expressed as mean ± SEMs. Means for two groups of data were compared using an unpaired *t* test. Means for groups were compared by analysis of variance followed by Tukey’s multiple comparison test. *P* values < 0.05 were considered statistically significant. All analyses were performed by an investigator blinded to the genotypes of the mice.

### Study approval

Experimental procedures of the human studies were approved by the Ethics Committee of Southeast University (approval number: 2018ZDKYSB042) and performed per the ethical standards laid down in the Declaration of Helsinki. All members of the family gave signed informed consent.

All mice-related experiments were per the Ethical Committee for Laboratory Animals (Southeast University) approval. Mice were housed under the care of the Animal Facility Interfaculty Unit, which is accredited by the Association for Assessment and Accreditation of Laboratory Animals. The experiments were performed on adult female HOM, HET, and WT mice.

## Results

### Clinical information on the family

**Fig. 2:**
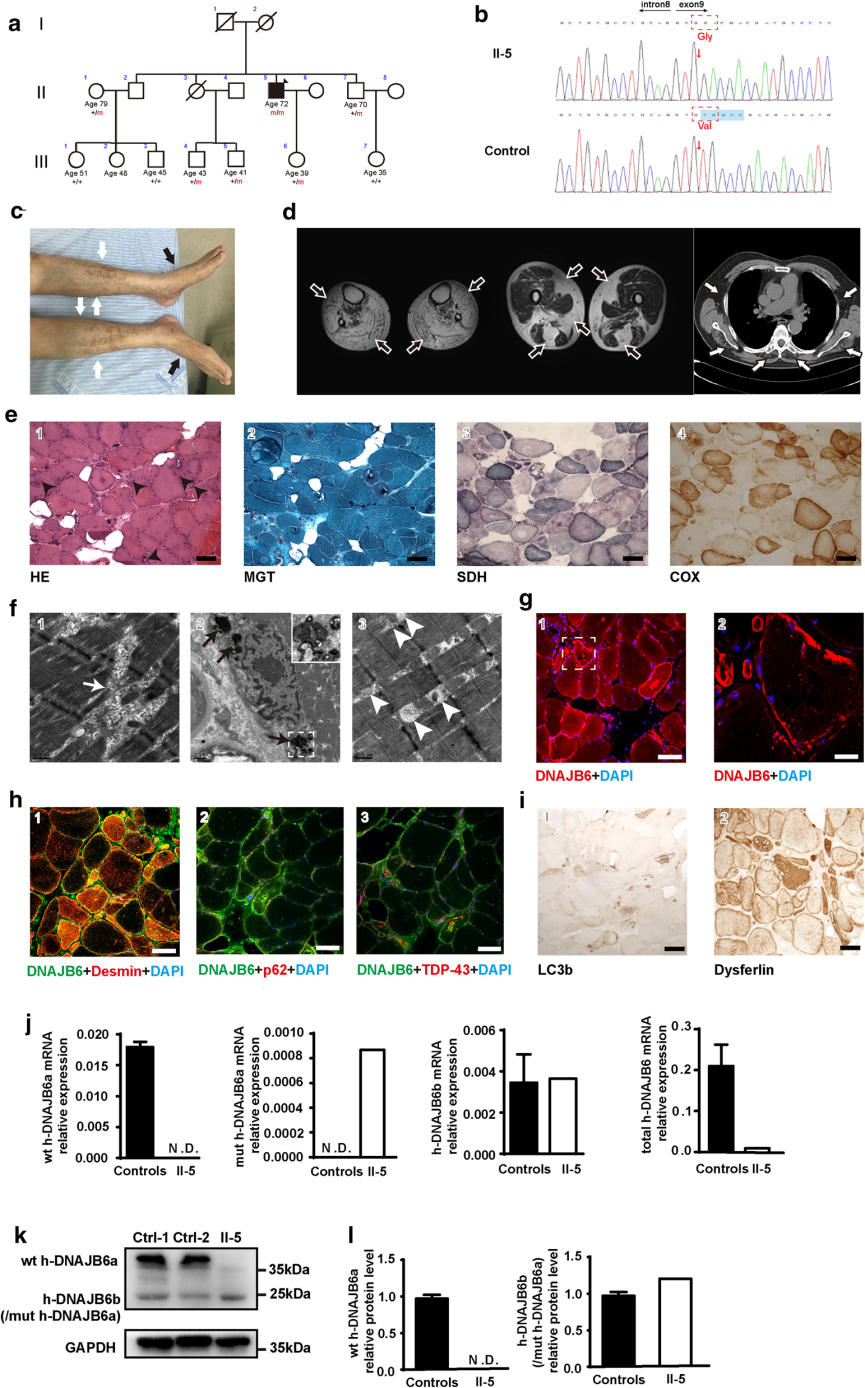
Clinical, histopathological and molecular features of the patient with the *DNAJB6* mutation. **a** The pedigree displayed co-segregation of the p.V232Gfs*7 homozygosis mutation (changes in the genotype labeled in red) with distal-onset myopathy. The arrow indicates the proband (individual II-5). Square: male; circle: female; open symbol: unaffected; filled symbol: affected; symbol with a diagonal line: deceased. **b** Representative chromatogram of the forward sequencing reaction in the proband displayed a novel mutation in *DNAJB6* genomic DNA (c.695_699del; p.V232Gfs*7) (the mutation of genome and amino acids labeled in red arrow and dotted frame). **c** Patient II5: distal lower limb atrophy (white arrows) and bilateral foot drop (black arrows). **d** MRI revealed generalized fatty replacement in the distal lower legs, and vastus, gracilis, and semitendinosus muscles (black arrows). Substantial muscle alterations with fatty infiltration were observed in paraspinal, infraspinatus, and intercostal muscles (white arrows). **e** Histochemical analysis of the vastus lateralis muscle biopsy demonstrated the presence of increased connective tissue, pathological variation of fiber diameter, pyknotic nuclear clumps, and rimmed vacuoles (black arrowheads), as shown in hematoxylin & eosin (H&E) staining. Modified Gomori trichrome (MGT) staining displayed sarcoplasmic masses located principally around the rimmed vacuoles. In addition, succinate dehydrogenase (SDH) and cytochrome C oxidase (COX) stains revealed multiple fibers with areas of diminished enzyme staining. Scale bar = 100 µm. **f** Electron microscopy indicated disruption of Z-disks (white arrows, 1), large electron-dense material located at the perinuclear regions (black arrows, 2), and abnormal mitochondria (white arrowheads, 3). Scale bars = 0.5 µm (1, 3), 1 µm (2). **g** Confocal microscopy showed that DNAJB6 staining highlighted in multiple fibers with subsarcolemmal accumulation and sarcoplasmic inclusions, while it was absent in myonucleus. Scale bars = 100 µm (1), 20 µm (2). **h** Confocal microscopy shows desmin co-located with DNAJB6 in muscle cytoplasm (1), p62 (2) and TDP-43 (3) strongly positive in rimmed vacuoles. Scale bar = 100 µm. **i** Immunohistochemical analysis showed LC3b staining around the rimmed vacuoles and Dysferlin accumulation in the cytoplasm. Scale bar = 100 µm. **j** RT-qPCR analysis of mRNA expression levels of human-wild type DNAJB6a (h-wt DNAJB6a), human-mutant DNAJB6a (h-mut DNAJB6a), human-DNAJB6b (h-DNAJB6b) and human-total DNAJB6 (h-total DNAJB6) in a muscle biopsy of individual II-5. **k** Representative Western blot analysis of muscle (30 µg) homogenates from controls and patient II-5, using DNAJB6 and GAPDH antibodies. Note that the h-DNAJB6b band might comprise h-mut DNAJB6a. **l** Relative quantification of the level of DNAJB6 in II-5 compared with controls demonstrates a clear reduction in DNAJB6. *P* value = * < 0.05, ** < 0.01

The family pedigree was consistent with an autosomal recessive inheritance pattern (Fig. 2a). The proband (patient II-5), a 72-year-old man born to non-consanguineous Chinese parents, achieved normal developmental milestones. He presented with an onset of weakness of ankle dorsiflexion and eversion at the age of 60 years. Symptoms of dyspnea coupled with progressive involvement of the proximal muscles were seen within 10 years after the initial onset. Upon examination, atrophy of the gastrocnemius and tibialis anterior muscle was observed bilaterally with foot drop (Fig. 2c), and lower limb weakness [proximal: Medical Research Council (MRC) 3/5; distal: MRC 2/5]. Reflexes were present in the upper limbs and knees but absent in the ankles. Sensation was intact. Nerve conduction studies revealed normal function. Electromyography studies revealed myopathic abnormalities in the deltoid, tibialis anterior, paraspinal, and vastus lateralis muscles.

Previous laboratory investigations indicated that CK levels ranged between 400 and 500 U/L (normal < 170 U/L). Cardiac examination (including electrocardiogram and echocardiogram) was normal. Pulmonary function tests revealed a moderate restrictive breathing disorder. MRI of the lower limbs indicated the presence of extensive fatty replacement in the distal lower limbs, with milder degenerative changes in the long head of the biceps femoris, semimembranosus, quadriceps femoris, adductor longus, and left adductor Magnus (Fig. 2d). Additionally, lung CT revealed fatty infiltrations in the intercostal, paravertebral, and subscapular muscles (Fig. 2d). Targeted exome sequencing was performed for the proband and his family members. Following the filtering of variants, a homozygous 5 base-pair deletion in the *DNAJB6* gene, c.695_699del (NM_058246), which localizes to exon 9, was found in the proband (Fig. 2b). The *DNAJB6* variant was not listed in the public database and was predicted to induce a frameshift from amino acid 232 (p. V232Gfs*7), leading to modification of the last 7 amino acids in addition to the 87 amino acids that are missing. Sanger sequencing confirmed the segregation of the mutation with the disease. None of the available healthy family members carried this homozygous mutation (Additional file 2: Figure S1).

Muscle biopsies of the right vastus lateralis (patient II-5) demonstrated MFM features with atrophy, hypertrophy, internal nuclei, fibrosis, fiber splitting, rimmed vacuoles, and sarcoplasmic masses (Fig. 2e 1-2). Fibers with a “moth-eaten” appearance were observed in SDH- and COX-stained sections (Fig. 2e, 3-4). Electron microscopy studies also demonstrated MFM features, such as focal disruption of the myofibrillar network, large electron-dense material located at the perinuclear regions, and abnormal mitochondria (Fig. 2f, 1-3). Protein aggregation was revealed using antibodies against DNAJB6, desmin, and dysferlin (Fig. 2 g, h-1, i-2). Rimmed vacuoles were strongly positive for TDP-43, p62, and LC3B (Fig. 2h 2-3, i-1). Additionally, DNAJB6 was observed in multiple fibers with subsarcolemmal accumulation and sarcoplasmic inclusions but was absent in myonuclei (Fig. 2g).

Surprisingly, compared to healthy controls, the level of total h-DNAJB6 mRNA (h-wt DNAJB6a + h-mut DNAJB6a + h-DNAJB6b) was 95% lower (Fig. 2j), and the level of its protein decreased by about 80% in patient (Fig. 2k-l). The mRNA level of DNAJB6b is similar between patient and health control (Fig. 2j). The data suggested that the mRNA and protein levels of DNAJB6a were largely reduced in the patient. Moreover, the level of desmin protein increased by about 50% (Additional file 2: Figure S2). These results suggested that MFMs might be caused by DNAJB6a insufficiency.

### *DNAJB6* c.698_702del knock-in mice: a model for human *DNAJB6* c.695_699del myopathies

**Fig. 3:**
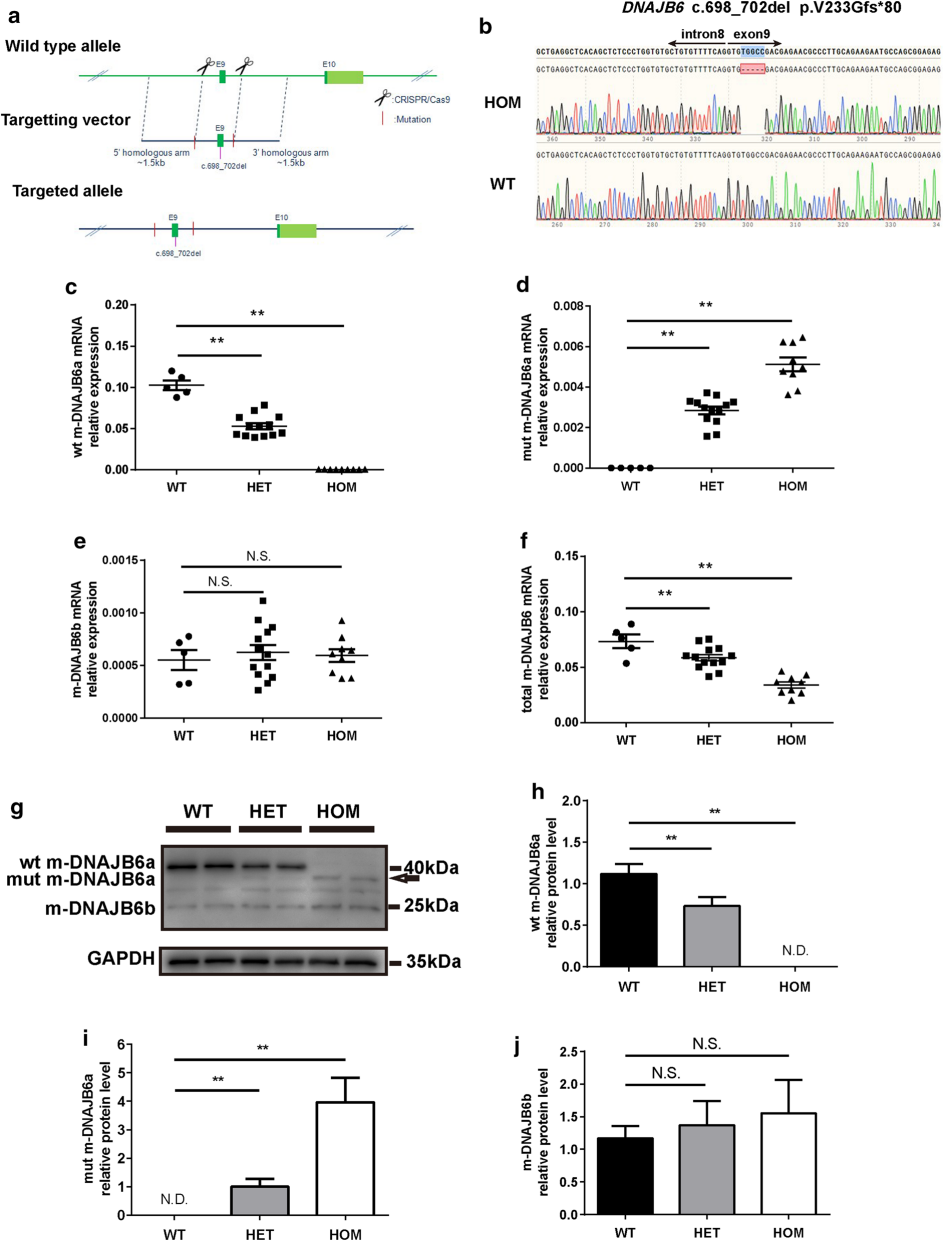
Generation and validation of m-*DNAJB6* c.698_702del KI mice. **a** Schematic representation of the KI mouse *DNAJB6* allele displaying the c.698_702del mutation flanked by two homology arms. **b** Sequencing of the m-*DNAJB6* gene demonstrating the presence of the mutation in a single allele of a homozygous (HOM) and wild-type (WT) animal. **c-f** RT-PCR analysis of mouse-wild type DNAJB6a (wt m-DNAJB6a), mouse-mutant DNAJB6a (mut m-DNAJB6a), mouse-DNAJB6b (m-DNAJB6b) and mouse-total DNAJB6 (total m-DNAJB6a) in the gastrocnemius muscle of HOM or heterozygous (HET) mice and WT littermates. Values are expressed as mean ± SEM (n = 5–13 per genotype). **g** Western blot analysis showing that the protein lysate from WT exclusively produced a wt m-DNAJB6a band, while HOM or HET mice produced an mutant m-dnajb6a band specifically (arrow). GAPDH was used as a loading control. **h-j** Quantification of relative intensities of proteins shown in **g**. Data represent mean ± SEM of 3 independent experiments (n = 3). *P* value = * < 0.05, ** < 0.01

The *DNAJB6* KI mouse lines were generated using a single targeting vector encompassing the c.698_702del mutation, integrated into exon 9 of the endogenous mouse *DNAJB6* gene. A gene-targeting construct containing the mutated exon 9 was developed, with a gene cassette downstream of exon 9 flanked by two homology arms (Fig. 3a). Male chimeras were used for germline transmission, and F0 mice were backcrossed to the C57BL/6 N line to generate a stable colony carrying the *DNAJB6* c.698_702del allele. HET mice were bred to generate HOM, HET, and WT littermates. Mouse genotypes were identified by Sanger sequencing to differentiate WT and HOM alleles (Fig. 3b). The 12-month-old HOM mice had significantly reduced mRNA and protein levels of DNAJB6a (not DNAJB6b) in the gastrocnemius muscle compared to those in the same staged WT mice (Fig. 3c-j), suggesting that the recessive mutation affected the transcription of DNAJB6a in muscles similar to that in humans.

The KI mice had normal behaviors such as feeding, grooming, and mating, which were comparable to those of WT mice (data not shown).

### *DNAJB6* KI mice develop a progressive defect in motor function

**Fig. 4:**
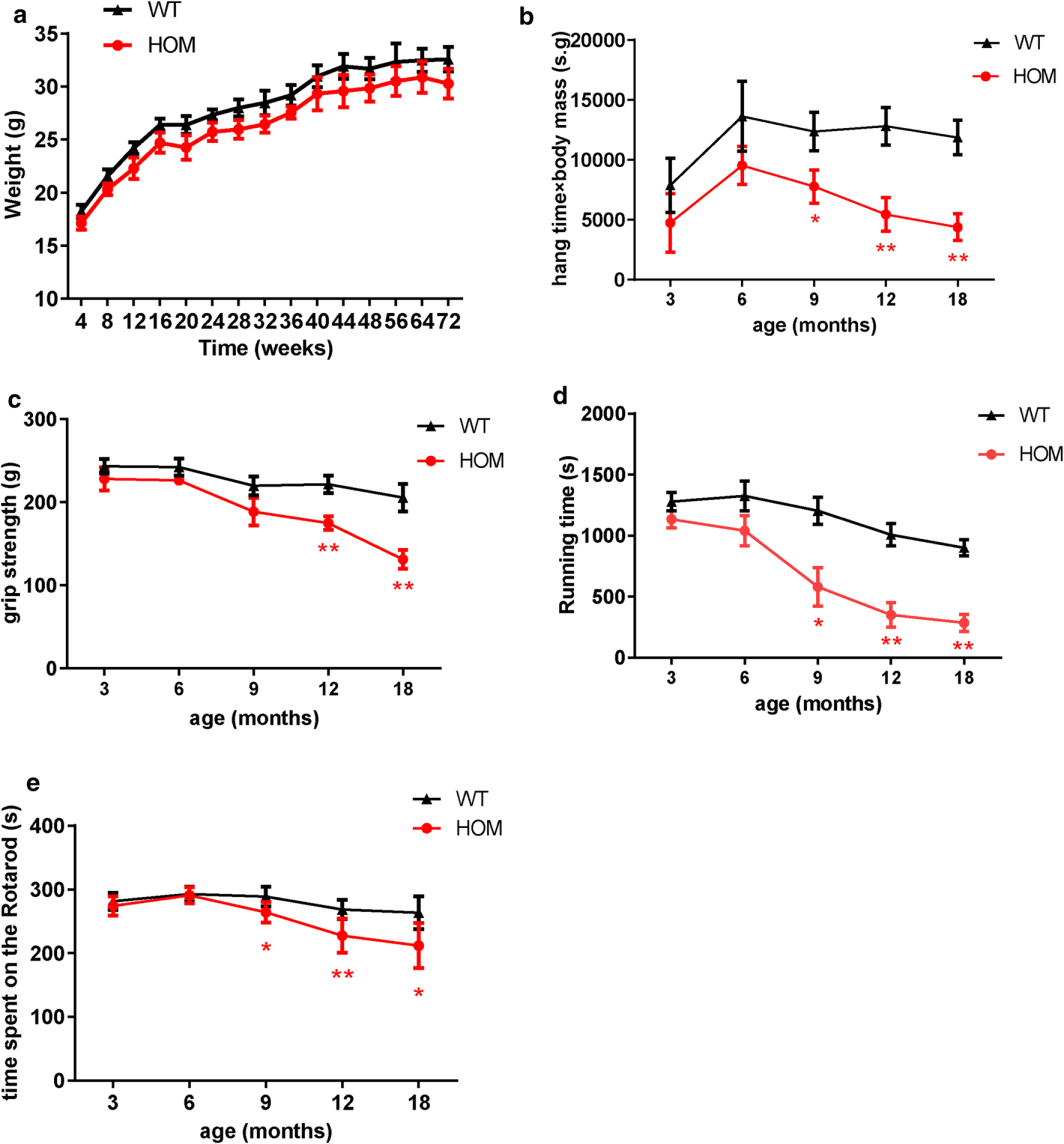
Assessment of motor function in the HOM and WT mice. **a** Body weight curves showing changes in body weight over 18 months for HOM and WT mice. Shown are mean ± SEM (n = 12–15) **b** Hanging test showing “minimal holding impulse” (body mass x hang time), in seconds (s) x gram (g), in 3, 6, 9, 12, 18-month old HOM mice and WT littermates. **c** Mean score in grip strength over time in HOM mice and WT littermates. **d** Treadmill experiments shown by duration of running, in seconds (s), before showing signs of exhaustion in HOM mice and WT littermates. **e** Rotarod performance showing mean duration, in seconds (s), spent on the accelerating rotating rod at 3, 6, 9, 12,18-month old HOM and WT mice. Values represent mean ± SEM (n = 12–15). *P* value = * < 0.05, ** < 0.01

There was no significant difference in body weights among the HOM, HET, and WT mice over the course of their development (Fig. 4a, Additional file 2: Figure S3a). The HOM, HET, and WT mice were screened for motor deficits at 3, 6, 9, 12, and 18 months of age. No differences in motor behaviors were detected among HOM, HET, and WT mice either at 3 or 6 months of age. After 9 months of age, the HOM mice displayed a progressive and significant decline in locomotor performance as assessed by the hanging, rotarod, and treadmill tests (Fig. 4b, d-e). A significant decline in grip strength was also observed after 12-months in HOM mice (Fig. 4c). Conversely, the performance of HET mice did not differ from that of WT animals (Additional file 2: Figure S3b-e). These data demonstrated that the *DNAJB6* c.698_702del recessive mutation induces late-onset motor behavioral impairment in mice.

### Structural pathology of skeletal muscle in *DNAJB6* KI mice

**Fig. 5:**
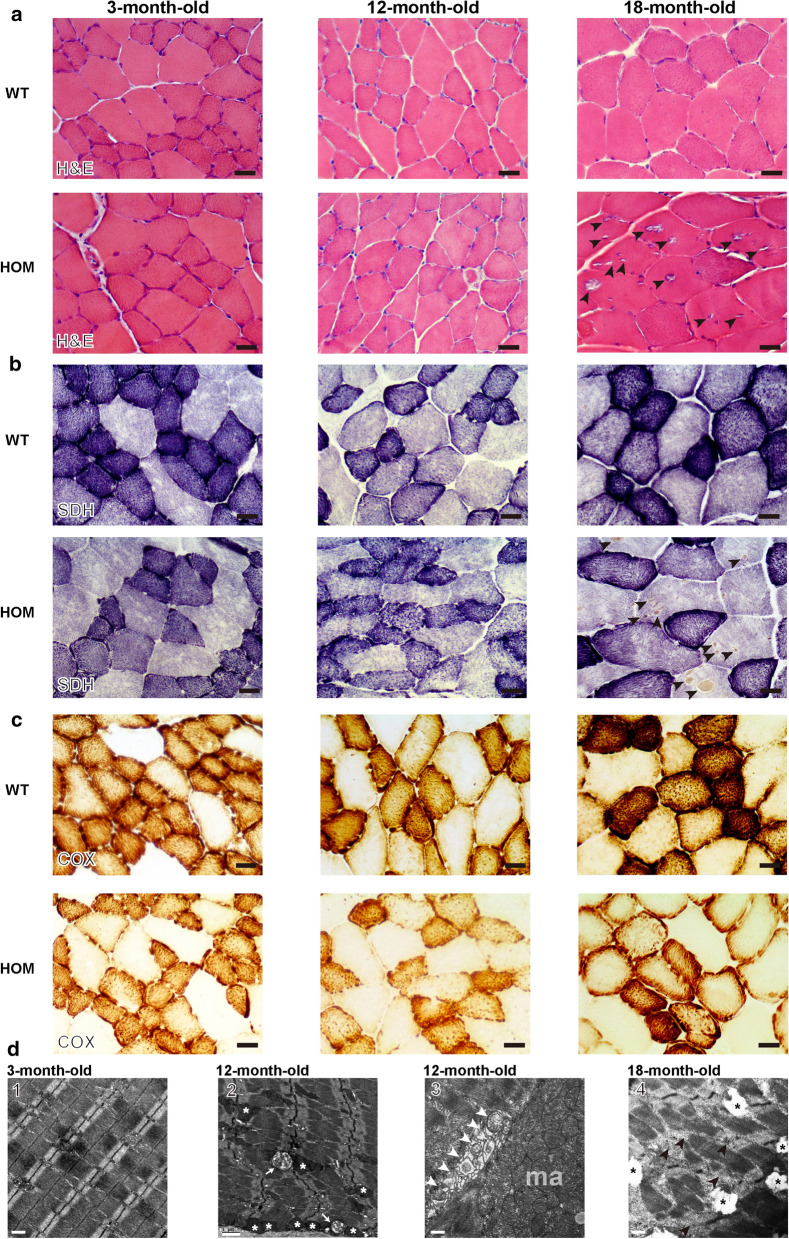
Tibialis anterior muscle histopathology of HOM and WT mice. **a**–**c** Representative HE (d), SDH (e) and COX (f) staining of tibialis anterior (TA) muscle from 3,12,18-month-old WT and HOM mice. Scale Bar = 100 µm. **d** Electron microscopy images of the TA muscle of 3-month-old HOM mice showed basically normal mitochondria regularly positioned adjacent to myofibrillar Z-discs (1). 12-month-old HOM mice displayed vacuolated (white arrow, 2) and enlarged mitochondria (white asterisk, 2) in the intermyofibrillar space, focal accumulations of mitochondria in the subsarcolemmal region (ma, 3), some of them undergoing mitophagy (white arrowheads, 3). 18-month-old HOM mice displayed severe myofiber disruption (black arrowheads, 4) and vacuolated mitochondria (black asterisk, 4). Scale bars = 0.5 µm (1, 3, 4), 1.0 µm (2)

To determine if the reduced motor function was associated with muscle degeneration, we examined the histology of the tibialis anterior (TA) muscles in *DNAJB6* KI mice at 3, 12, and 18 months of age. Myopathological analysis showed that the myofibers of 3-month-old HOM and WT mice were of regular size, with normal SDH and COX enzymatic activities (Fig. 5a–c). In contrast, 12-month-old HOM mice, but not their WT littermates, displayed rubbed-out areas with attenuated COX and SDH enzymatic activities (Fig. 5a–c), indicating the focal depletion of mitochondria in multiple muscle fibers. In aged TA muscle of 18-month-old mice, mitochondrial abnormalities were even more pronounced with multiple fibers displaying absent COX enzymatic activities and sarcoplasmic mass aggregates based on SDH staining. Notably, H&E staining showed abundant rimmed vacuoles in myofibers (Fig. 5a). Furthermore, the myopathic phenotypes of different staged HOM mice were refined by EM analyses, in which 3-month-old HOM mice showed basically normal mitochondria, regularly positioned adjacent to myofibrillar Z-discs (Fig. 5d), whereas enlarged and vacuolated mitochondria in the intermyofibrillar and subsarcolemmal regions were present in the muscles of 12-month-old HOM mice (Fig. 5d). Moreover, severe myofiber disruption and vacuolated mitochondria were observed in 18-month-old HOM mice (Fig. 5d). These data on aged-mice were similar to the MFM-like phenotype in our proband (patient II-5). However, HET mice displayed no overt myopathic alterations (data not shown). These data suggest that the *DNAJB6* c.698_702del recessive mutation causes progressive myopathy with distinct myofibrillar and mitochondrial abnormalities in mice.

### Muscle fiber type composition in *DNAJB6* KI mice

**Fig. 6:**
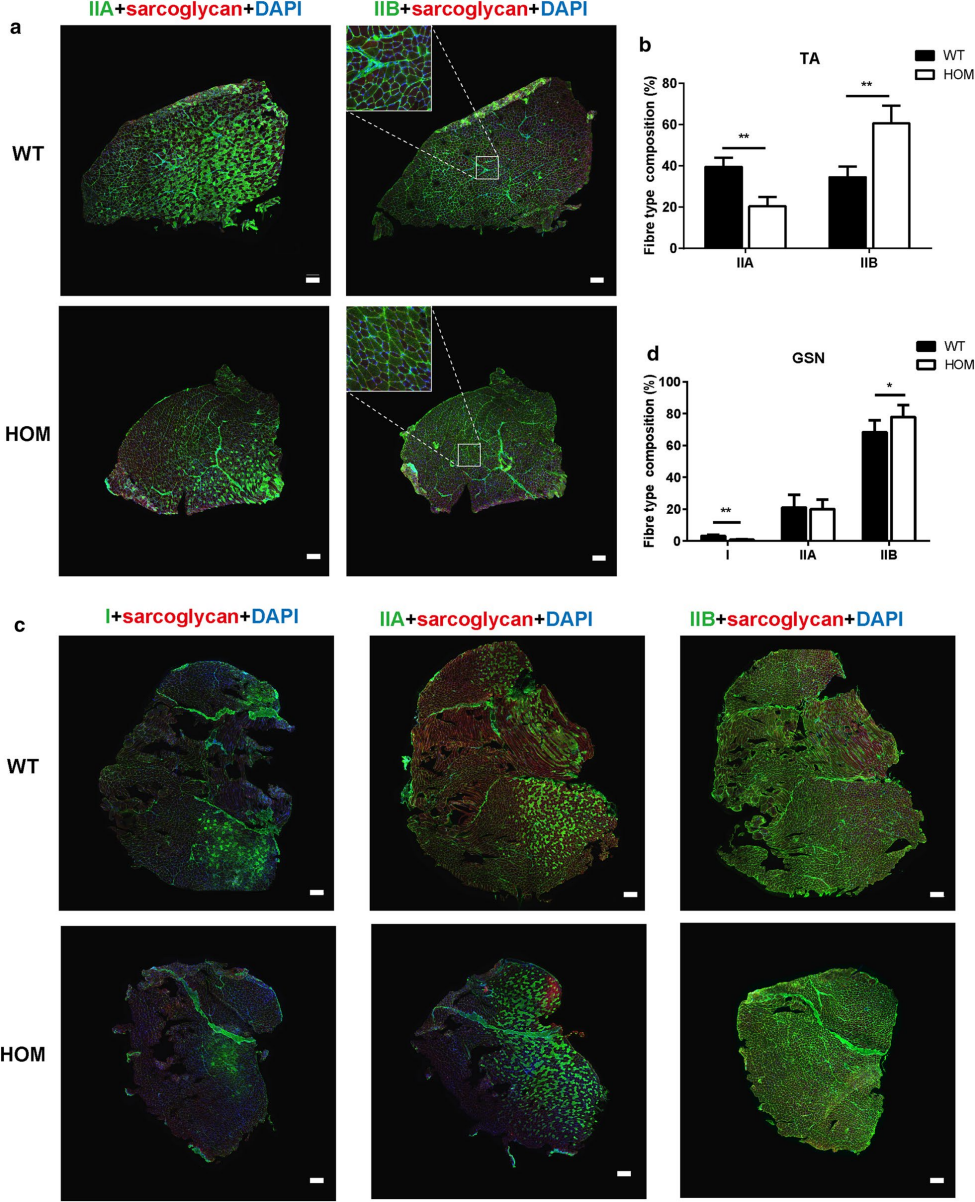
Changes in fiber type composition of tibialis anterior and gastrocnemius muscles in 12-month-old HOM and WT mice. **a, b** Representative transverse sections of tibialis anterior (TA) (a) and gastrocnemius (GSN) (b) muscles from 12-month-old WT and HOM mice immunostained with fiber type-specific myosin heavy chain antibodies. Scale bar: 200 µm. Note the decrease in proportion of type IIA fibers and increase in type IIB fibers in the TA from HOM mice (**a**), and the decrease in type I fibers in parallel with an increase in type IIB fibers in the GSN from HOM mice (**b**). **c, d** Quantified fiber type distribution by percent of total in individual fiber types. Values are expressed as mean ± SEM (n = 3). *P* value = * < 0.05, ** < 0.01

To analyze the full-field view of fiber type composition in various muscles, sections of the TA and gastrocnemius (GSN) muscles were immunostained with antibodies against specific myosin heavy chain isoforms, and the images were acquired and stitched together using laser point-scanning confocal microscopy. Representative images displayed in Fig. 6 demonstrate a substantial shift towards fast fiber types in both the TA and GSN muscles in 12-month-old HOM mice. Specifically, the proportion of type IIA fibers was reduced by 48%, whereas the proportion of IIB fibers was 1.5 to twofold greater in the TA muscles of HOM mice (Fig. 6a–b). The basal expression of type I myofibers was sparse in TA, and their expression was not altered in HOM mice (data not shown). The GSN muscles of the HOM mice showed a 60% decrease in the proportion of type I fibers, whereas the proportion of type IIB fibers was significantly higher (Fig. 6c–d). These findings indicated that the *DNAJB6* c.698_702del recessive mutation causes a substantial shift in fiber type composition towards fast fibers in mice.

### Aggregation of myofibril components in skeletal muscle in *DNAJB6* KI mice

**Fig. 7:**
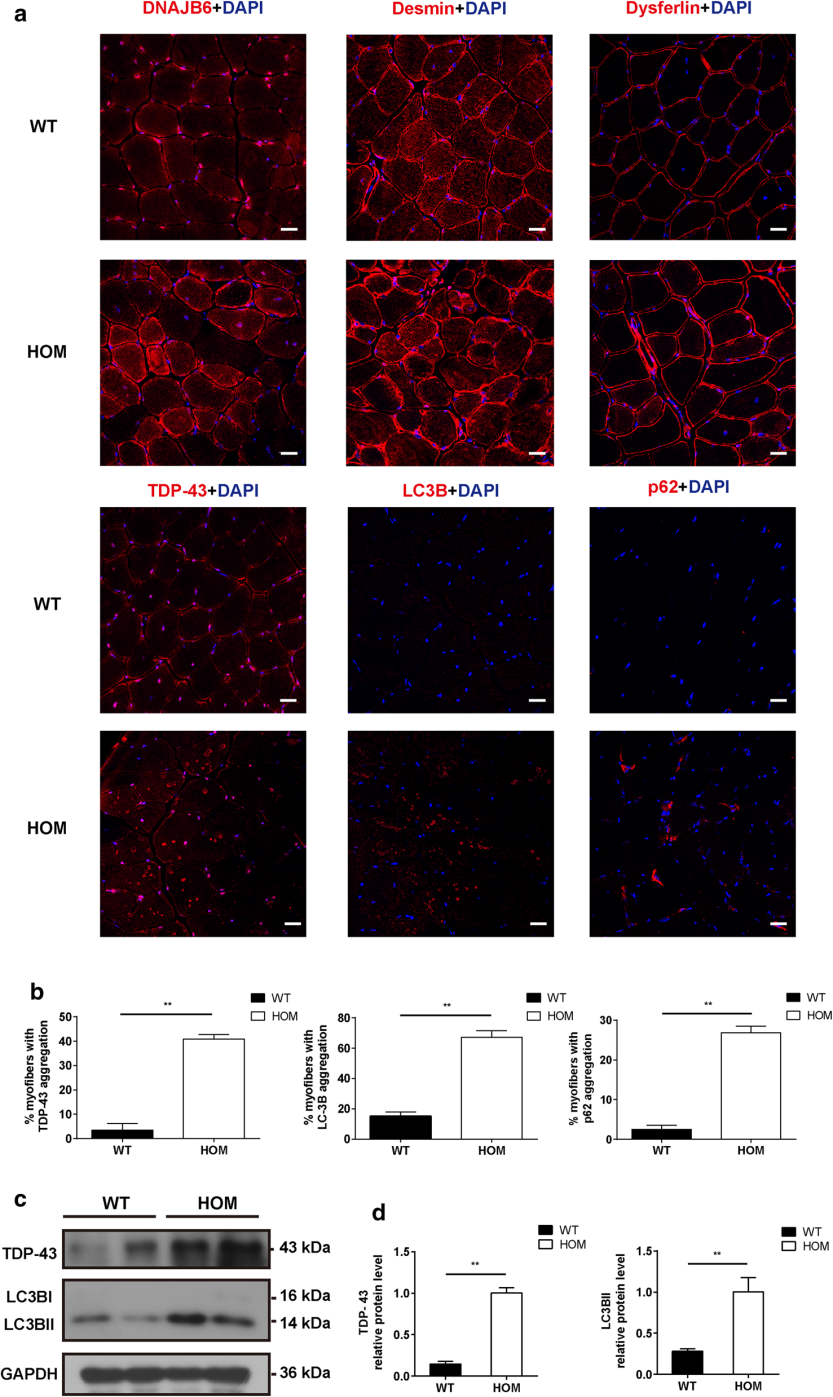
Aggregation of myofibril components in skeletal muscle tissue of 18-month-old HOM and WT mice. **a** DNAJB6, desmin, dysferlin, TDP-43, LC3B and p62 staining displayed altered subcellular distribution in the HOM mice. Scale bar = 20 µm. **b** Percentage of myofibers with TDP-43, LC3B and p62 aggregation in 18-month-old HOM and WT mice. **c** Western blot showing TDP-43 and LC3B in muscle lysates of 18-month-old HOM and WT mice. GAPDH was used as a loading control. **d** Quantification of relative intensities of proteins shown in **c**. Data represent means ± SEM of 3 independent experiments (n = 3). *P* value = * < 0.05, ** < 0.01

Since the aggregation of myofibrillar or ectopic proteins and high reactivity for several markers of proteasome impairment or autophagic defects are the major hallmarks of DNAJB6-related MFMs [36], immunofluorescence for DNAJB6, desmin, dysferlin, TDP-43, LC3B, and p62 was performed on the TA muscle of 3-, 12-, and 18-month-old mice. Compared to those on age-matched WT and HET mice, DNAJB6 immunostaining patterns in HOM mice exhibited markedly reduced fluorescence signal intensities in the muscular nucleus. The signal of DNAJB6 and desmin in the sarcoplasmic regions was uniform in the 3-month-old mice (Additional file 2: Figure S4a-b). In addition, aberrant DNAJB6 and desmin distributions were observed in 12- and 18-month-old HOM mice, with multiple fibers displaying large sarcoplasmic and subsarcolemmal aggregates (Fig. 7a, Additional file 2: Figure S4a–b). The desmin level was significantly higher in 12-month-old HOM mice than that in WT mice (Additional file 2: Figure S4c-d). Furthermore, dysferlin protein showed mild ectopic expression in part of the muscle fibers in 18-month-old HOM mice (Fig. 7a). As expected, the immunofluorescence experiments suggested that the muscle fibers from 18-month-old HOM mice exhibited much more TDP-43, LC3B, and p62 expression (Fig. 7a–b). Moreover, these proteins were concentrated around the rimmed vacuoles or in the sarcoplasmic regions, in concordance with the muscle immunohistochemical pathologic distinction found in the proband (Fig. 7a). Western blot analysis of the GSN tissue lysates also demonstrated a significantly increased amount of TDP-43 and LC3B II in HOM mice (Fig. 7c–d). This suggested that the *DNAJB6* c.698_702del recessive mutation in mice causes myofibril component aggregation in the muscles.

### A dose–effect relationship in the prevention of PolyQ aggregation of mutant DNAJB6a within cells

**Fig. 8:**
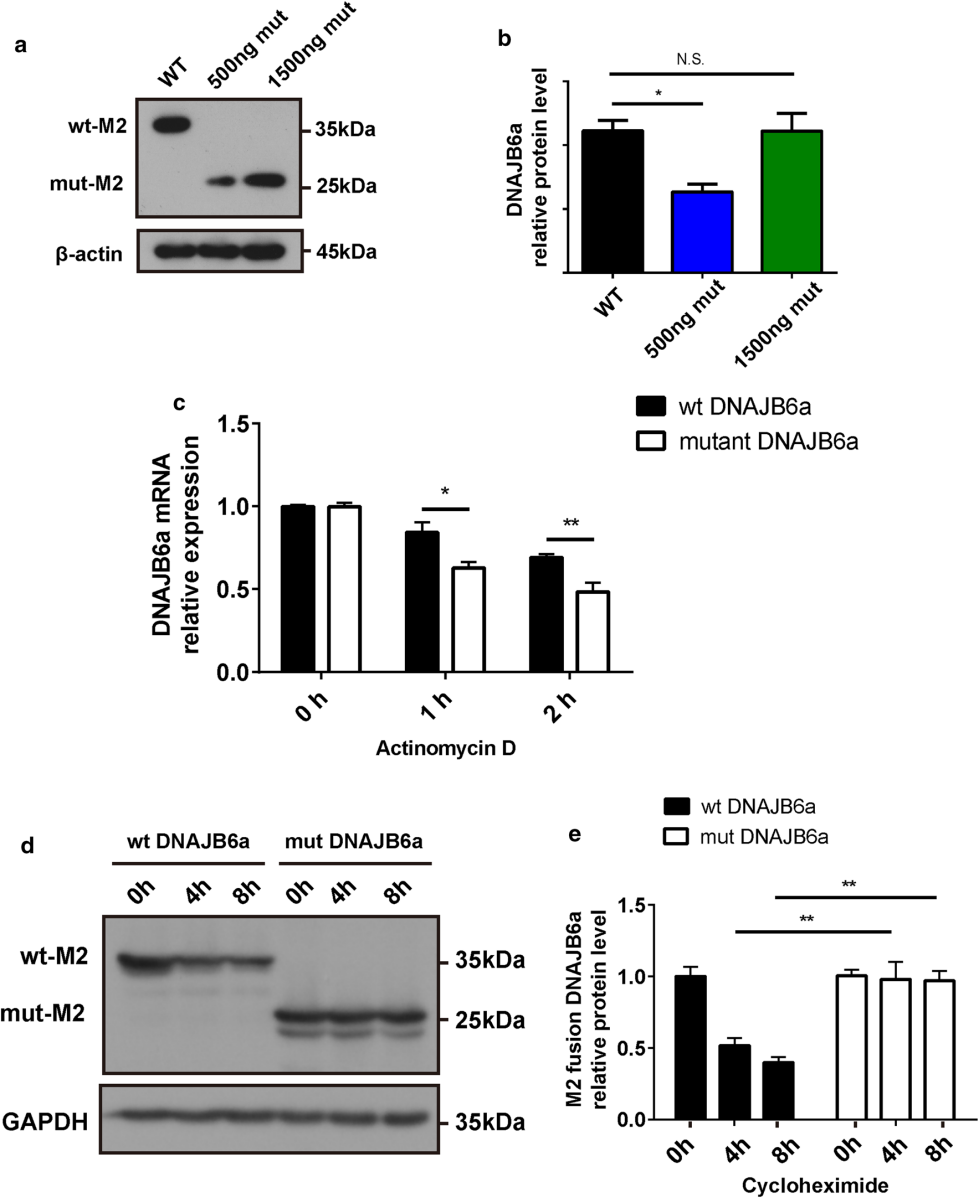
Effects of the c.695_699del recessive mutation in *DNAJB6* on its mRNA and protein stabilities in cells. **a** Lysates from HEK293 cell extracts transfected with 500 ng wild type M2-fused DNAJB6a (WT), 500 ng or 1500 ng M2-fused mutant DNAJB6a (500 ng mut, 1500 ng mut) plasmids, followed by immunoblot analysis with antibodies against M2 and β-actin. **b** Quantification of data in **a**. Data from three experiments (mean ± SEM). **c** RT-PCR analysis of WT or mutant DNAJB6a constructs transferred in HEK293 cells after actinomycin D (5 ug/ml) treatment for the indicated times points. **d** Western blot analysis of M2-fused WT or mutant DNAJB6a protein in HEK293 cells followed the treatment of cycloheximide (10 ug/ml) for the indicated time points. e Quantification of data in **d**. Data from three experiments (mean ± SEM). *P* value = * < 0.05, ** < 0.01. N.S. = not significant

To understand the effects of the novel recessive mutation, we first tested the distribution of GFP-tagged WT or mutant constructs in HEK293 cells. Only the WT constructs present in the nucleus exclusively, whereas the mutant DNAJB6a localizes to either the nucleus or the cytosol (Additional file 2: Figure S5). Furthermore, the levels of M2-tagged DNAJB6a were investigated using western blot analysis. 500 ng mutant DNAJB6a construct (27 kDa) were detected to have much lower protein levels in HEK293 cells than 500 ng WT construct (36 kDa) (Fig. 8a–b). The findings also revealed that similar levels were expressed with the administration of 500 ng for WT and 1500 ng for mutants (Fig. 8a–b).

To address whether the insufficient expression of mutant DNAJB6a is due to reduced mRNA stability or protein stability, we transfected WT and mutant DNAJB6a expression vectors into HEK293 cells. As a result, the mRNA transcribed from the mutant construct degraded more rapidly than the mRNA from the WT construct after actinomycin D (5 ug/ml) treatment (Fig. 8c). However, cycloheximide (10 ug/ml) treatment rapidly decreased WT DNAJB6a protein levels, whereas the mutant DNAJB6a proteins showed reduced turnover (Fig. 8d–e). These data demonstrated that the *h*-*DNAJB6* c.695_699del recessive mutation decreased the level of mutant DNAJB6a protein via reducing the mRNA stability.

**Fig. 9:**
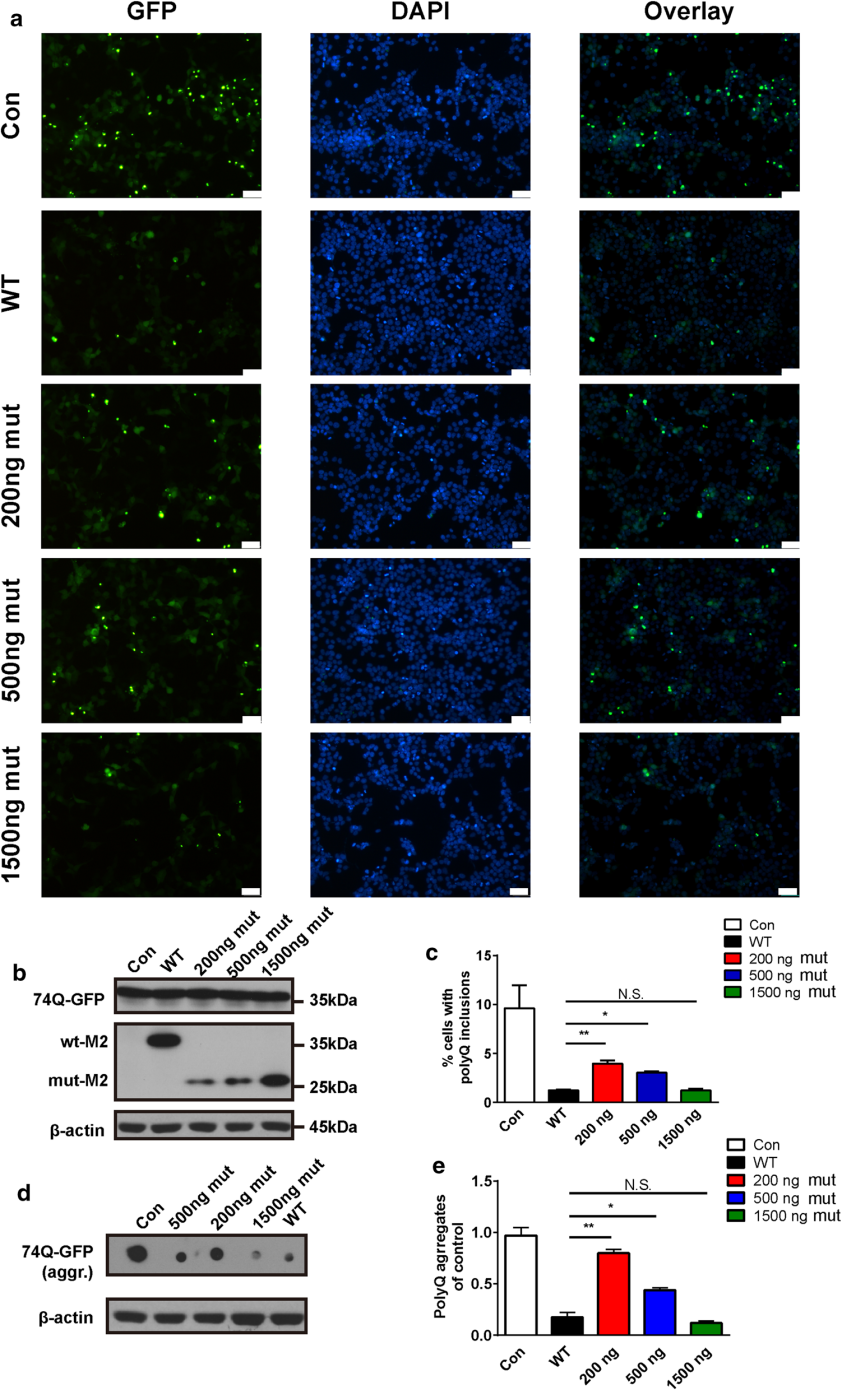
Effects of the c.695_699del recessive mutation in *DNAJB6* on its ability to prevent aggregation of polyQ in cells. **a** Representative immunofluorescent images of HEK293 cells cotransfected with HttEx1-(Q)74-hrGFP and 500 ng empty vector as control (Con), 500 ng wild type M2-fused DNAJB6a (WT), 200 ng, 500 ng or 1500 ng mutant M2-fused DNAJB6a (200 ng mut, 500 ng mut, 1500 ng mut) plasmids respectively for 48 h. Scale bar = 50 µm. **b** Western blot showing Lysates from cells treated as in **a.** with antibodies against GFP, M2, and β-actin. **c** Quantitative analysis of **a**, expressing the proportion of cells containing inclusions (each scored by at least three independent observations and then averaged). **d** Filter-trap assay. Cell lysates in **a.** were loaded onto cellulose acetate membranes and probed with an anti-GFP antibody to detect aggregation of HttEx1-(Q) 74-hrGFP. **e** Quantification of data in **d**. Data from three experiments (mean ± SEM). *P* value = * < 0.05, ** < 0.01. N.S. = not significant

To explore the anti-aggregation capability of human mutant DNAJB6a within cells, the inhibition of aggregation of expanded polyQ proteins was analyzed in a cell model. Immunofluorescence analysis was conducted on HEK293 cells co-transfected with *pcDNA3.1*-*HttEx1*-*(Q)74*-*hrGFP* and WT or different doses of mutant *DNAJB6* constructs (200 ng mut, 500 ng mut and 1500 ng mut) or empty vectors (Con) (Fig. 9a–b). Cells expressing only HttEx1-(Q) 74-hrGFP proteins displayed numerous nuclear aggregates, which were dissolved into a diffuse pattern when cells co-expressed WT DNAJB6 and 74Q proteins (Fig. 9a–c). However, the 74Q proteins still formed multiple nuclear aggregates when cells co-expressed different doses of mutant DNAJB6 and 74Q proteins (Fig. 9a–c). This finding indicates that the same quantity of WT and mutant DNAJB6a protein has an equivalent effect on anti-aggregation of the expanded 74Q proteins, and this capability progressively reduced with a decreasing dose of mut-DNAJB6a.

A filter-trap assay was performed to further estimate the anti-aggregation activity of the WT or mutant DNAJB6a proteins. As expected, 1500 ng mutant plasmid was necessary to have the same effect on aggregations as 500 ng WT plasmid, which suggested the mutant DNAJB6a protein had anti-aggregation activity with similar efficiency to the same quantity of WT DNAJB6a protein (Fig. 9d–e), and displayed a dose-dependent relationship with the inhibition of aggregation of 74Q, consistent with the results of immunofluorescence analysis (Fig. 9a–c).

## Discussion

In the present study, we first reported a novel homozygous frameshift mutation of *DNAJB6* in the proband of a Chinese family segregating a recessive MFM. Intriguingly, the novel recessive mutation resulted in a decrease in DNAJB6a levels, but not DNAJB6b. A knock-in model of the equivalent variant in mice resulted in a recessive myopathy with late-onset reduction in muscle function similar to the patient. The variant expressed in HEK293 cells, showed a dose dependent effect in the prevention of polyQ aggregation.

Many published studies have focused on the structure–function relationships of DNAJB6 [16, 19–21, 26, 42]. The J-domain is believed to be the domain for DNAJ-Hsp70 interactions [20], whereas the G/F domain plays a crucial role in substrate processing (refer to Fig. 1) [42]. Recently, the S/T region has been confirmed to have efficient anti-aggregation properties against polyQ-containing or amyloid-containing proteins, independent of Hsp70 [16, 26, 47]. Additionally, Karamanos et al. revealed that the “b” region (Fig. 1) could affect the oligomerization state of DNAJB6b and the entire fold of the protein [21]. Unexpectedly, the novel mutation found in the present study, uniquely causing the loss of the “a” region (Fig. 1), resulted in a significant reduction in mutant DNAJB6a mRNA and truncated DNAJB6a protein levels both in human and mouse. In light of the fact that the mutation localizes to the 5′ region of exon 9, and the mut DNAJB6a mRNA stability was reduced, we speculated that DNAJB6a mRNA harboring the premature-translational-termination-codon mutation is degraded through the nonsense-mediated mRNA decay pathway and thus leads to a considerably lower level of the truncated protein [29].

To date, all reported myopathic mutations have been located in exons 4 and 5, which affect both isoforms of DNAJB6 [8, 17, 18, 22, 28, 30, 31, 35, 37, 39, 45]. A recent study showed that LGMD D1 mutations in *DNAJB6* have a dominant gain-of-function effect on protein quality control and myopathic phenotypes, which can be partially corrected by inhibiting the DNAJB6–HSP70 interaction in both yeast and mouse models [3]. However, DNAJB6b-F93L overexpressing mice exhibit obvious myopathic features compared to DNAJB6a-F93L overexpressing mice, which indicated that the DNAJB6b-mutant isoform was the pathogenic driver of myopathy [4]. Intriguingly, we found a novel recessive mutation, c.695_699del (p.V232Gfs*7) in *DNAJB6*, associated with a late-onset distal MFM. Notably, the mutation led to a decrease in DNAJB6a levels, but not DNAJB6b. Furthermore, Hageman et al. identified DNAJB6a to be as effective as DNAJB6b on polyQ antiaggregation in a cell model [16]. In addition, zebrafish deficient in the DNAJB6a ortholog show increased cardiac endoplasmic reticulum (ER) stress, whereas DNAJB6a overexpression inhibits ER stress in zebrafish and protects mice from doxorubicin-induced cardiomyopathy [11]. Recently, deletion of DNAJB6 in myoblasts resulted in myofibrillar disintegration and sarcomeric protein accumulation [13].Given these results, we propose that loss of DNAJB6a protein might lead to the cause of MFM in our reported proband.

Indeed, the *DNAJB6* c.698_702del (corresponding to the human *DNAJB6* c.695_699del mutation) knock in (KI) mice developed progressive weakness, myofiber disorganization, desmin accumulation and striking rimmed vacuoles in muscles, which were the MFM features reported in our proband. Although the length of the mutant truncated DNAJB6a in KI mice (311 amino acids) was different from that predicted in the proband (237 amino acids), the amino acid sequences after the mutant amino acid were not homologous to those of the wide-type DNAJB6a, and the levels of mutant DNAJB6a mRNA and protein were exceedingly low. Thus, we speculated that this recessively inherited MFM we reported might be due to loss of DNAJB6a protein, not a gain-of-function effect of truncated mutant DNAJB6a protein. Furthermore, the *DNAJB6* c.698_702del mouse model is essential for understanding the pathogenic mechanism of DNAJB6a in the development of MFM

In this mouse model, we found that the level of mut DNAJB6a in HOM mice was significantly lower than the level of wt DNAJB6a in WT mice, with a reduction of more than 50%, whereas the level of DNAJB6a (mut DNAJB6a + wt DNAJB6a) in HET mice remained higher than 50% of the levels in WT mice. Therefore, we speculate that half of DNAJB6a (wt DNAJB6a or mut DNAJB6a) is sufficient to maintain myofibril integrity and function. However, a decline in DNAJB6a levels down to a specified threshold could impair protein quality control in skeletal muscle, leading to aggregation-prone protein accumulation and cytotoxicity. The proposed mechanism is further supported by cellular experiments in which the anti-aggregation capability decreased progressively with a decreasing dose of mut-DNAJB6a. The diplo-insufficiency mechanism also explains why the variant that we identified appears to be recessive.

The clinical phenotype of *DNAJB6* c.695_699del patient was recessive and late-onset, with symptoms starting in the distal lower limb weakness. With advanced age, however, the symptoms involved also respiratory muscles and proximal muscles, causing more disability. These phenotypes were not consistent with that caused by other variants identified in *DNAJB6*, which were associated with dominant, more severe disease [28, 30]. It is common that mutations in MFM-causing genes can result in different phenotypes, ranging from normal to severely disabled, even within the same family [23], mutations in *DNAJB6* are not an exception on this. Genetic and epigenetic factors, as modifying disease severity, might play roles on the heterogeneity of clinical phenotypes.

In conclusion, our findings broaden the mutational spectrum of *DNAJB6*-related MFMs. Further, the novel recessive mutation led to the loss of the C-terminal part of DNAJB6a and caused myopathic characteristics of MFMs in humans and mice, probably via a loss of function in suppressing cytotoxic protein aggregation of DNAJB6a.

## Supplementary information

**Additional file 1: Table**.

**Additional file 1: Figures**.
