# Supplementary material for: A novel recessive mutation affecting DNAJB6a causes myofibrillar myopathy
Source: Acta Neuropathol Commun. 2021 Feb 8;9:23. doi: 10.1186/s40478-020-01046-w (PMC7869515; doi:10.1186/s40478-020-01046-w)
Supplement: Supplementary file 1 — Additional file 1: Table. [file 40478_2020_1046_MOESM1_ESM.pdf]

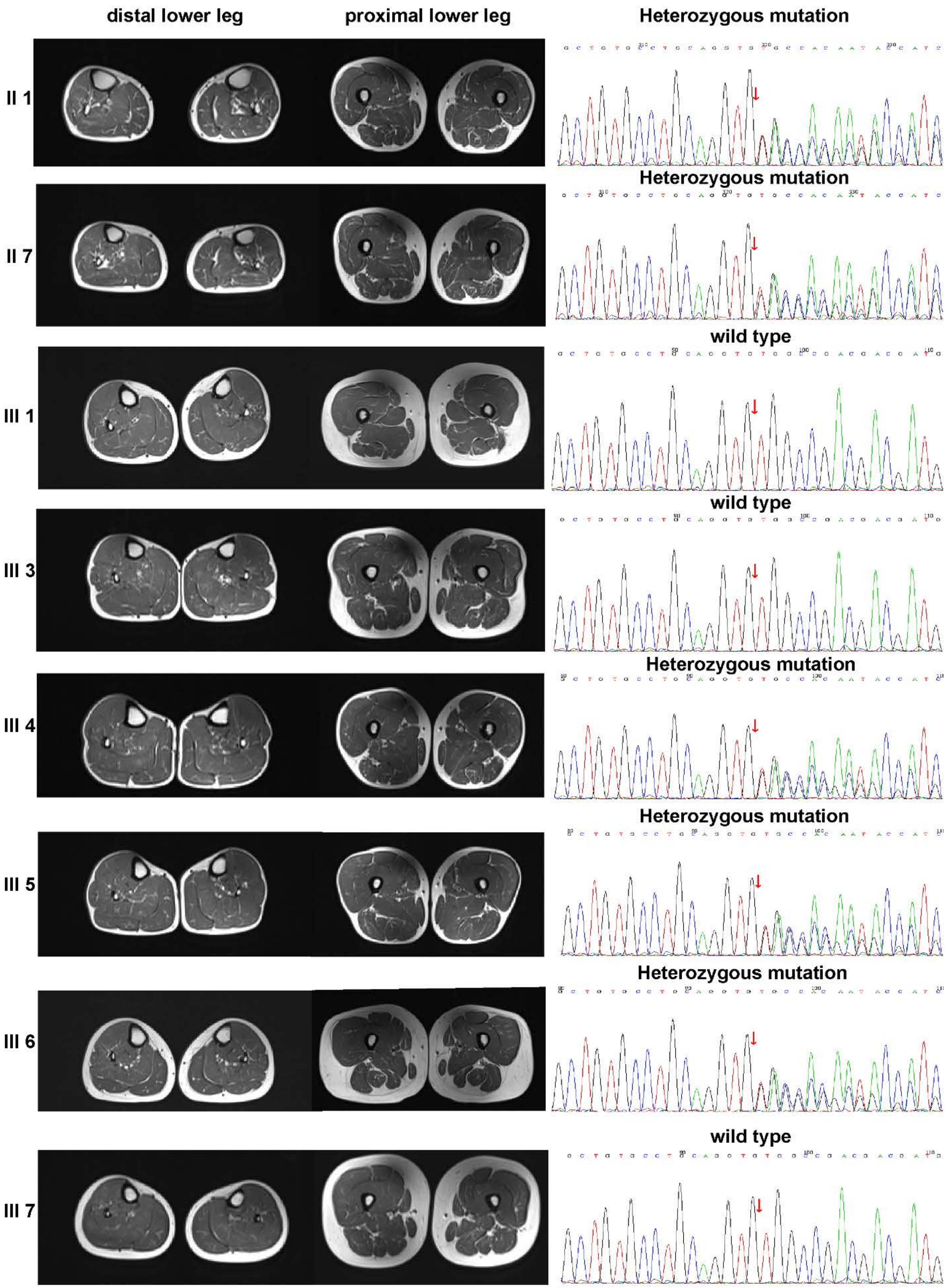

distal lower leg
proximal lower leg
Heterozygous mutation
II 1
Heterozygous mutation
II 7
wild type
III 1
wild type
III 3
Heterozygous mutation
III 4
Heterozygous mutation
III 5
Heterozygous mutation
III 6
wild type
III 7

Figure S1 Muscle MRI imaging and representative chromatogram of forward sequencing reaction of the proband's family members. No apparent signs of abnormality in the muscle of proximal and distal lower limbs.

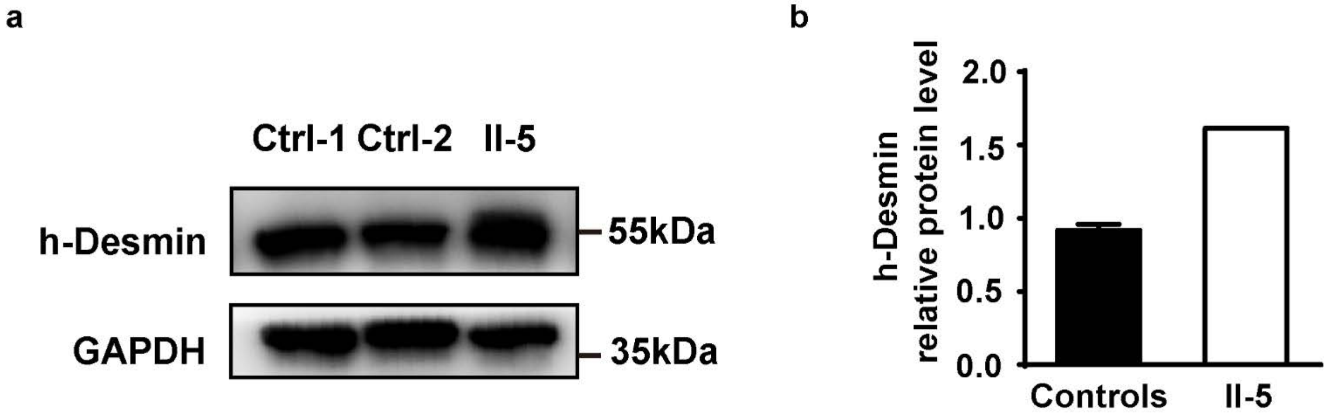

Figure S2 Western blot analysis of desmin protein in the patient with the *DNAJB6* mutation. **a** Representative Western blot analysis of muscle (30 µg) homogenates from controls and patient II-5, using desmin and GAPDH antibodies. **b** Quantification of relative intensities of proteins shown in **a**. P value=*<0.05, **<0.01.

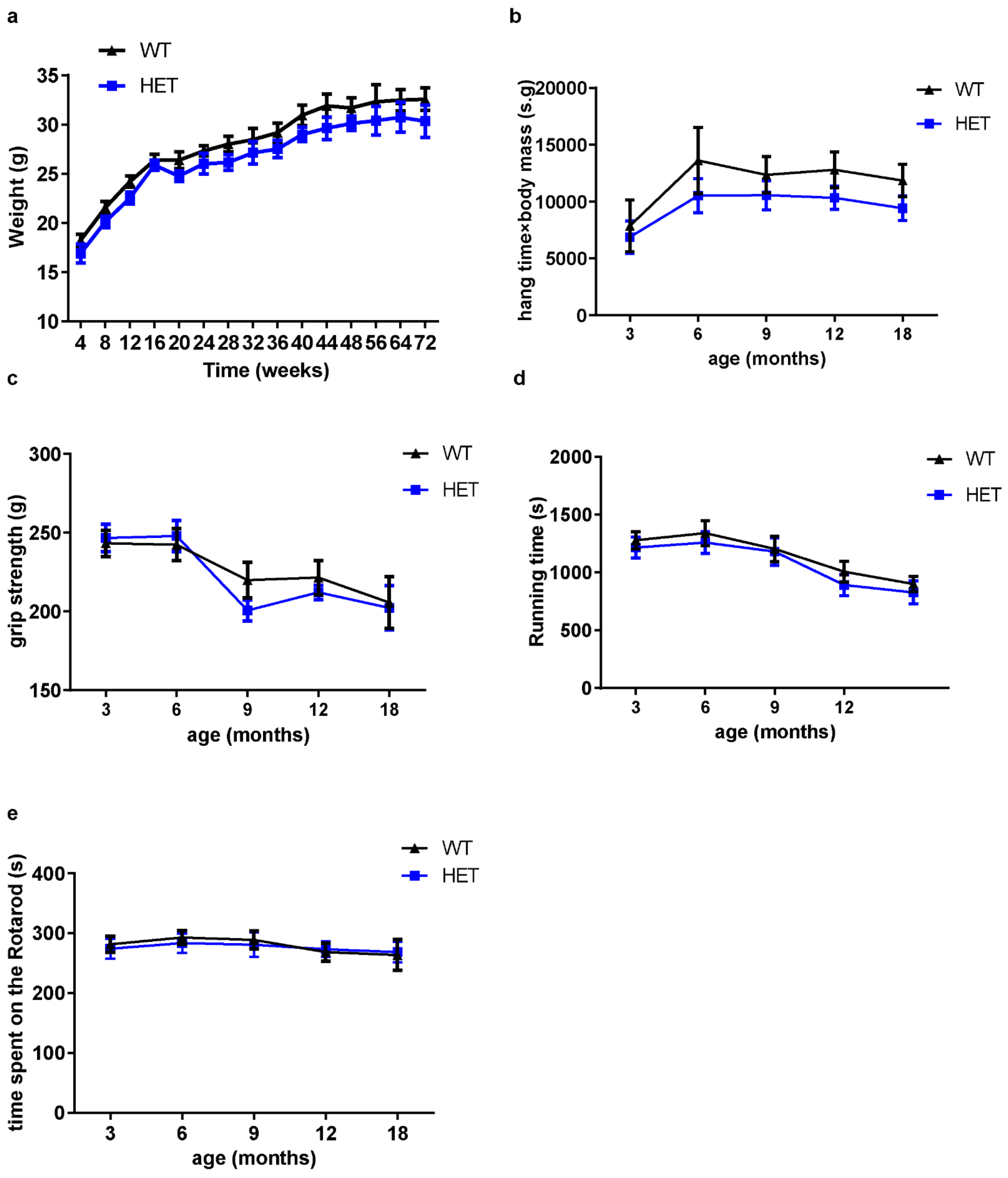

Figure S3 Assessment of motor function in the HET and WT mice. **a** Body weight curves showing changes in body weight over 18 months for HET and WT mice. Shown are mean ± SEM (n = 12-15) **b** Hanging test showing "minimal holding impulse" (body mass x hang time), in seconds (s) x gram (g), in 3, 6, 9, 12, 18-month old HET mice and WT littermates. **c** Mean score in grip strength over time in HET mice and WT littermates. **d** Treadmill experiments shown by duration of running, in seconds (s), before showing signs of exhaustion in HET mice and WT littermates. **e** Rotarod performance showing mean duration, in seconds (s), spent on the accelerating rotating rod at 3, 6, 9, 12,18-month old HET and WT mice. Values represent means ± SEM (n = 12-15). P value=*<0.05, **<0.01.

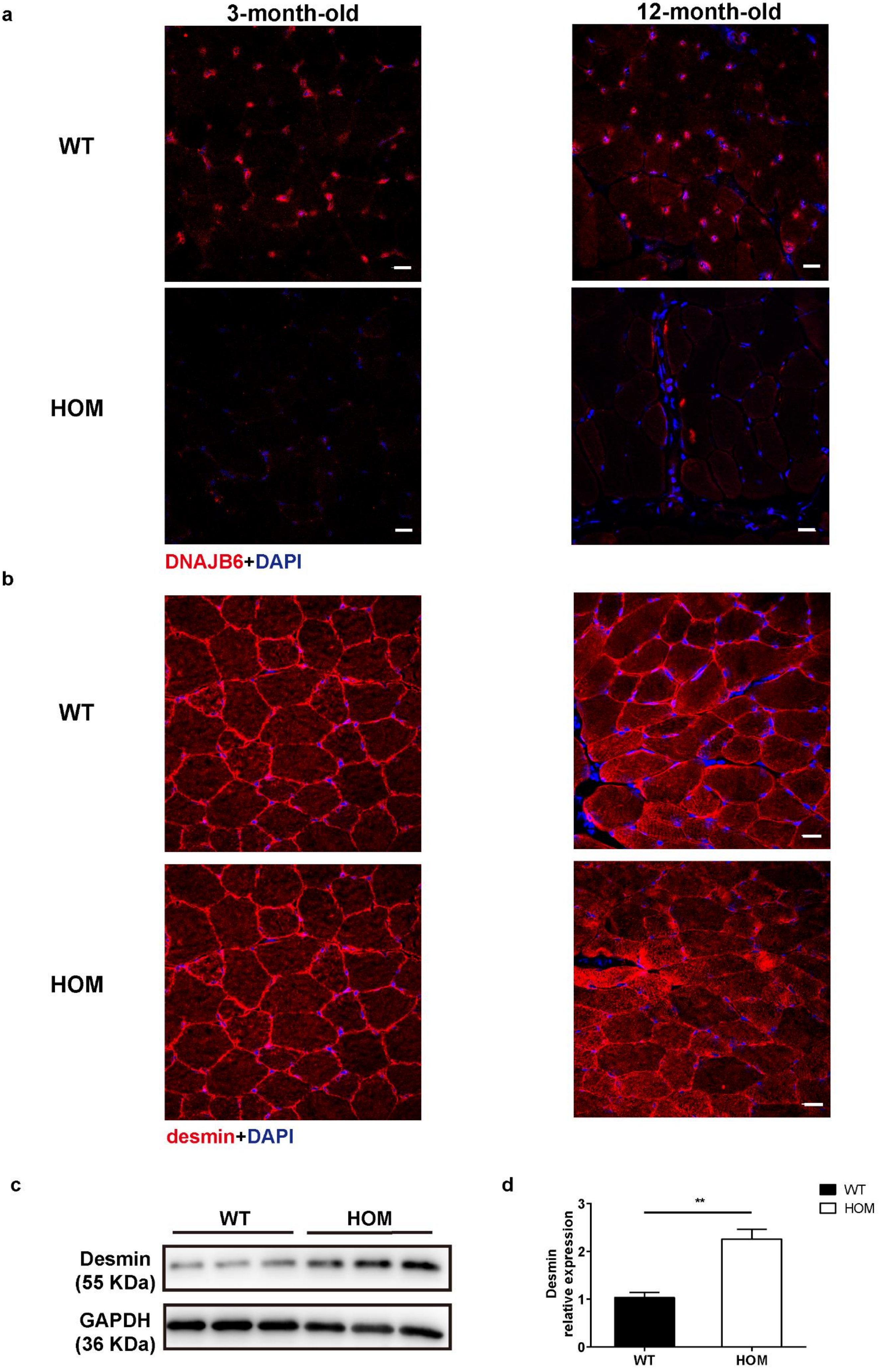

a
3-month-old
12-month-old
WT
HOM
DNAJB6+DAPI
b
WT
HOM
desmin+DAPI
c
WT
HOM
Desmin
(55 KDa)
GAPDH
(36 KDa)
d
WT
HOM
Desmin
relative expression
**
0
1
2
3
WT
HOM

Figure S4 Subcellular distribution of DNAJB6 and desmin in skeletal muscle tissue of 3 and 12-month-old HOM and WT mice. **a** DNAJB6 staining displayed diminished nuclear staining of muscle fibers in HOM mice compared with WT mice, while no accumulation in the sarcoplasm in the 3-month-old mice. 12 -month-old HOM mice displayed sarcoplasmic and subsarcolemmal aggregates in some muscle fibers. Scale bar = 20 μm. **b** Desmin was uniformly stained in the sarcoplasm of muscle fibers in HOM mice when 3 months old, while displayed sarcoplasmic and subsarcolemmal aggregates when 12 months old. Scale bar = 20 μm. **c** Western blot analysis of gastrocnemius muscle (30 μg) homogenates from 12-month-old WT and HOM mice, using desmin and GAPDH antibodies. **d** Quantification of relative intensities of proteins shown in **c**. Data are shown as means ± SEM of 3 independent experiments (n = 3). P value=*<0.05, **<0.01.

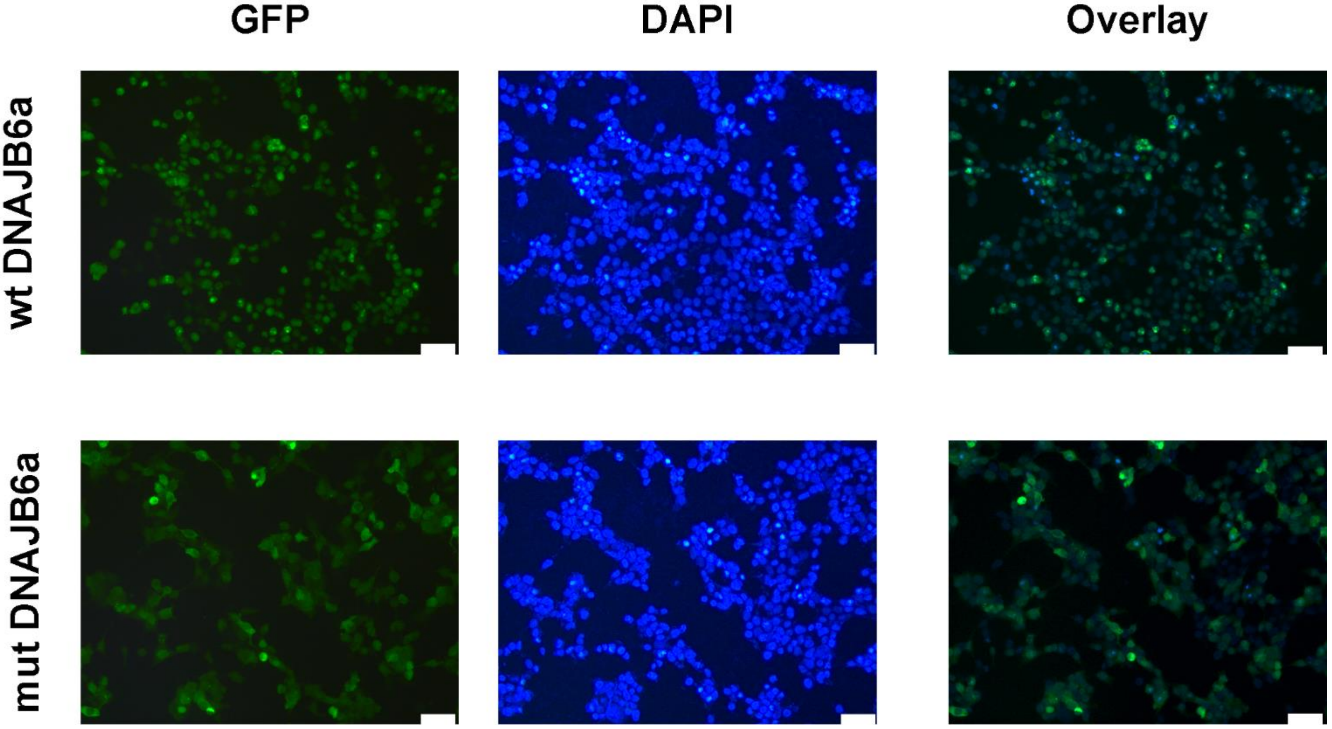

Figure S5 The distribution of wild type DNAJB6a and mutant DNAJB6a in HEK293 cells. **a** Representative immunofluorescent images of HEK293 cells transfected with 500ng wild type GFP-fused DNAJB6a (wt DNAJB6a) plasmids for 48h. Scale bar = 50 μm. **b** Representative immunofluorescent images of HEK293 cells transfected with 1500ng mutant GFP-fused DNAJB6a (mut DNAJB6a) plasmids for 48h.Scale bar = 50 μm.
