# Supplementary material for: A novel recessive mutation affecting DNAJB6a causes myofibrillar myopathy
Source: Acta Neuropathol Commun. 2021 Feb 8;9:23. doi: 10.1186/s40478-020-01046-w (PMC7869515; doi:10.1186/s40478-020-01046-w)
Supplement: Supplementary file 2 — Additional file 1: Figures. [file 40478_2020_1046_MOESM2_ESM.docx]

**Table S1** Specific primer sequences of *DNAJB6* and *GAPDH* in human and mouse

| Primer | Forward (5’-3’) | Reverse (5’-3’) |
| --- | --- | --- |
| *wt h-**DNAJB6a* | TTGGTGGTAGTGGCATGGGC | ATCGTCGTCGGCCACACCATT |
| *mut h-DNAJB6a* | TTGGTGGTAGTGGCATGGGC | GGCATCGTCGTCCACCATTT |
| *h-DNAJB6b* | TTGGTGGTAGTGGCATGGGC | GCAGCAGCTGCTCCTTACCATT |
| *total h-DNAJB6* | CATGCCTCACCCGAGGATATT | CCTCCGCTACTTGCTTGAATTT |
| *wt m-DNAJB6a* | TCATTTGGCGGCAGTGGA | GTTCTCGTCGGCCACACCATTTA |
| *mut m-DNAJB6a* | TTGATACAGGCTTCACTCCATT | GCGTTCTCGTCCACCATT |
| *m-DNAJB6b* | GGGGACTTCCATTTTCAGGC | CGTCACTCAGGGCGTCTTCTA |
| *total m-DNAJB6* | TGAAAATAAAGAAGAAGCAGAGC | AAGAAGTCAAATGAAAATGGGT |
| *m-GAPDH* | AAGAAGGTGGTGAAGCAGG | GAAGGTGGAAGAGTGGGAGT |
| *h-GAPDH* | GGACCTGACCTGCCGTCTAG | GTAGCCCAGGATGCCCTTGA |

Note: wt, wild type; mut, mutant; h, human; m, mouse.
